# Malaria parasite egress at a glance

**DOI:** 10.1242/jcs.257345

**Published:** 2021-03-08

**Authors:** Michele S. Y Tan, Michael J. Blackman

**Affiliations:** 1 Malaria Biochemistry Laboratory, The Francis Crick Institute, London NW1 1AT, UK; 2 Faculty of Infectious and Tropical Diseases, London School of Hygiene & Tropical Medicine, London WC1E 7HT, UK

**Keywords:** *Plasmodium*, Protozoan, Malaria, Egress

## Abstract

All intracellular pathogens must escape (egress) from the confines of their host cell to disseminate and proliferate. The malaria parasite only replicates in an intracellular vacuole or in a cyst, and must undergo egress at four distinct phases during its complex life cycle, each time disrupting, in a highly regulated manner, the membranes or cyst wall that entrap the parasites. This Cell Science at a Glance article and accompanying poster summarises our current knowledge of the morphological features of egress across the *Plasmodium* life cycle, the molecular mechanisms that govern the process, and how researchers are working to exploit this knowledge to develop much-needed new approaches to malaria control.

**Figure:**
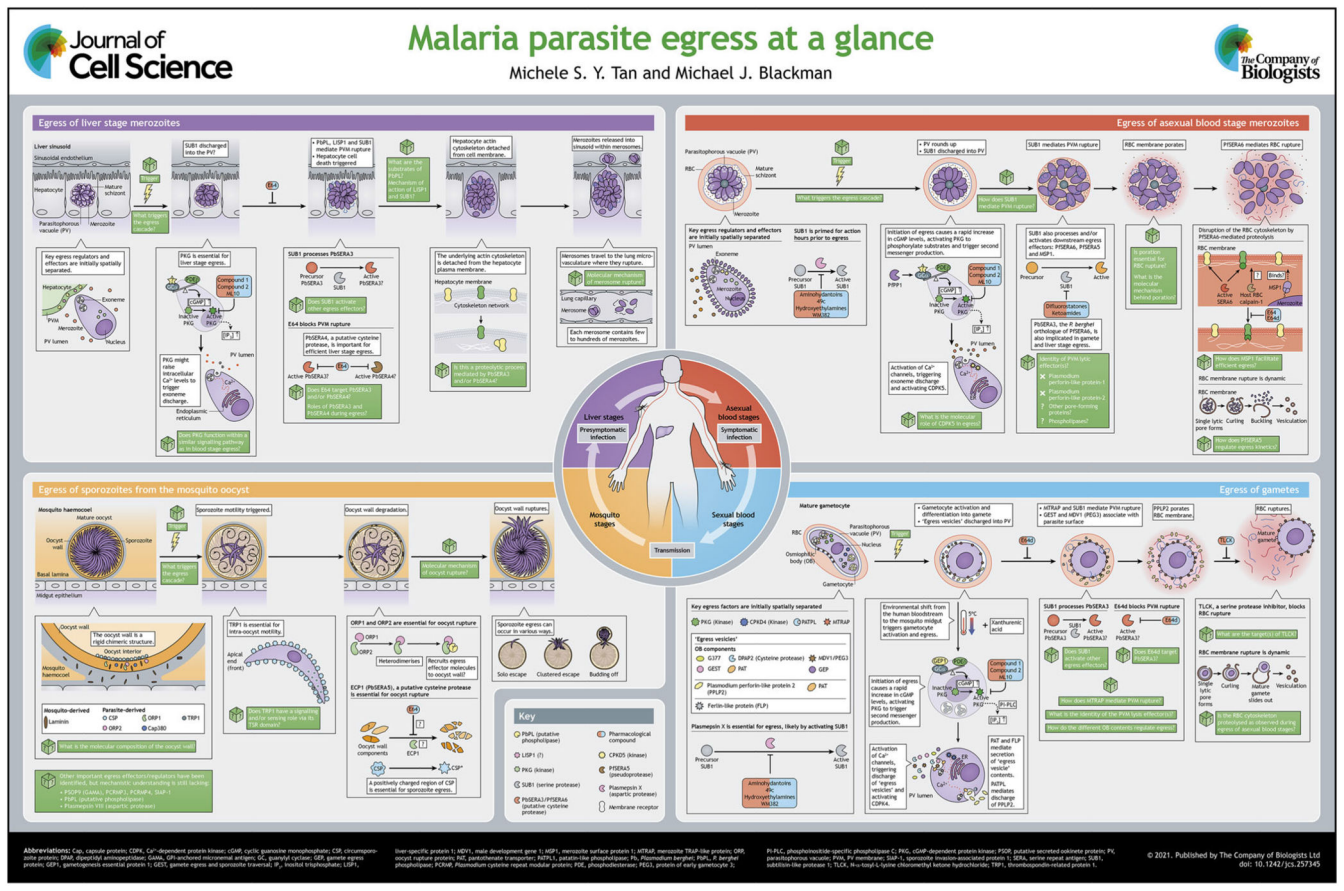

## Introduction

Malaria is a devastating infectious disease that impacts over 30% of the world’s population, causing over 400,000 deaths annually (World Health Organization, 2020). The causative agents, species of the genus *Plasmodium,* belong to a phylum of predominantly obligate intracellular protozoan parasites called the Apicomplexa, which includes numerous other pathogens of clinical and veterinary importance, such as the agents of toxoplasmosis, cryptosporidiosis, coccidiosis, babesiosis and theileriosis (Seeber and Steinfelder, 2016). Although much of the biology of these organisms is unique to each genus or even species, a unifying feature is that replication takes place only within a cyst or the intracellular environment of an infected host cell. As a result, the replicative parasite life cycle stages are interspersed by the release (egress) and dissemination of invasive and often motile forms called zoites that seek out and actively invade specific cells of their hosts. Malaria transmission takes place only via an insect vector, the female *Anopheles* mosquito. The parasite life cycle spans both the vertebrate host and insect vector (see Box 1), so parasite egress must occur in these very different environments. Despite this, it has become increasingly clear over the past two decades that the molecular mechanisms controlling egress – while distinct – share some remarkably similar regulatory and/or effector molecules, allowing development of unifying models for the signalling and effector pathways involved (Friedrich et al., 2012; Wirth and Pradel, 2012; Flieger et al., 2018). As our knowledge of egress increases, we predict that these parallels may become increasingly evident.

The malaria parasite replicates within three distinct settings: the mosquito oocyst, the vertebrate hepatocyte, and the vertebrate red blood cell (RBC). Details vary between the six different *Plasmodium* species that cause malaria in humans, including the stage of RBC maturation preferred. Here, we describe current knowledge of egress and highlight outstanding questions in the field. We focus primarily on *Plasmodium falciparum* (denoted by the prefix Pf where necessary), the most deadly species, but, where relevant, reference is also made to other *Plasmodium* species, including the widely used model rodent species *Plasmodium berghei* (prefix Pb); this species also has certain biological similarities to *Plasmodium vivax,* which is the most important agent of malaria outside of Africa but cannot be maintained *in vitro*, hindering its experimental analysis.

## Sporozoite egress from the mosquito oocyst

The oocyst is surrounded by a poorly defined structure called the oocyst wall or capsule, which ruptures to release sporozoites. Despite many previous electron microscopy (EM) studies of oocyst structure and egress (e.g. Sinden and Strong, 1978; Orfano et al., 2016), details of the dynamics of sporozoite egress have come to light only recently (see poster). Using an elegant *ex vivo* time-lapse light microscopy approach, egress has been seen to take place in several superficially distinct manners (Klug and Frischknecht, 2017). Unexpectedly, escaping sporozoites sometimes appear to initially bud into large vesicle-like protrusions that emerge from a single point of rupture of the oocyst capsule. In all cases, egress is preceded by extensive circular movement of the sporozoites within the oocyst.

Reverse genetic studies have implicated several parasite gene products in sporozoite egress, although in only a few cases have these shed light on their mechanistic role. A seminal example of this was examination of the cysteine protease-like protein ECP1 (a ‘group I’ SERA; see Box 2). ECP1-null sporozoites developed normally and displayed the intra-oocyst motility mentioned above, but failed to egress (Aly and Matuschewski, 2005). Importantly given the presumed proteolytic role of ECP1, the authors noticed that a characteristic western blot signal produced upon probing oocyst extracts with antibodies to the major circumsporozoite protein (CSP) was more complex in the ECP1 mutant, suggesting altered CSP proteolysis. As well as being localised to the sporozoite surface, CSP is a component of the inner layer of the oocyst wall (Thathy et al., 2002) so it is significant that the mutant oocysts are insensitive to detergent permeabilisation, in contrast to wild-type oocysts, and relatively resistant to mechanical stress (Aly and Matuschewski, 2005). Collectively, these observations suggest an essential role for ECP1 in proteolytic degradation of the oocyst wall at egress. However, ECP1 has yet to be demonstrated to possess protease activity.

Gene disruption has similarly implicated the putative aspartic protease plasmepsin VIII (PMVIII) in egress. PMVIII-null sporozoites lack motility and fail to egress from oocysts (Mastan et al., 2017). A study of the α-thrombospondin repeat (TSR)-containing putative integral membrane sporozoite protein TRP1 has shown that, similar to ECP1 and PMVIII, its loss results in sporozoites that develop normally but fail to egress (Klug and Frischknecht, 2017). TRP1-null sporozoites display no intra-oocyst motility, so it is surprising that mechanically released TRP1-null sporozoites show no motility defect, leading the authors to speculate that TRP1 plays a role in activating sporozoite motility prior to egress (Klug and Frischknecht, 2017). TRP1 could play a signalling or sensing role, as TSR domains are often involved in protein–protein interactions (Adams and Tucker, 2000). However, as shown by the ECP1 mutant described above, sporozoite motility is clearly not sufficient for egress.

Other players identified as important for sporozoite egress include the two related histone-fold domain proteins oocyst rupture protein 1 (ORP1) and ORP2 (Currá et al., 2016). ORP1 is located to the capsule, whereas ORP2 initially localises throughout the oocyst body. Remarkably, both relocalise to the oocyst wall around the time of sporozoite egress, probably forming a heterodimer (Currá et al., 2016) (see poster). Sporozoites lacking ORP1 or ORP2 develop normally and are motile if mechanically released, but do not undergo egress (Currá et al., 2016). Despite detailed mutagenesis analysis of the twin histone-fold domains of ORP2 (Siden-Kiamos et al., 2018), how the ORP proteins contribute mechanistically to oocyst rupture remains unknown. Several other proteins, including CSP, PCRMP3 and PCRMP4 (Douradinha et al., 2011), PSOP9 (also known as GAMA) (Ecker et al., 2008), SIAP-1 (Engelmann et al., 2009) and the putative phospholipase PbPL (Burda et al., 2015), are also important for egress. The pathways disrupted in the respective mutants are likely diverse. For example, CSP-null mutants do not form sporozoites at all (Ménard et al., 1997), possibly due to the loss of this abundant glycolipid-anchored protein from the plasma membrane of the developing syncytium (Wang et al., 2005b); however, more subtle alterations of CSP that do not block protein expression also prevent egress (Wang et al., 2005a). With regard to these other factors, PbPL likely acts to hydrolyse oocyst phospholipids, but PSOP9 and SIAP-1 possess no recognisable domains and so, as is the case for ORP1 and ORP2, their mechanistic role is not understood. Future progress in understanding sporozoite egress will require a clearer picture of the molecular composition and fate of the oocyst capsule at egress, and will be aided by the availability of an increasing number of egress-defective parasite mutants and improvements in oocyst isolation (e.g. Siden-Kiamos et al., 2020).

## Egress of liver stage merozoites

Studying the development of liver stage parasites is challenging, in part due to the very small proportion of hepatocytes that become parasitised even under optimal conditions. However, several technically outstanding intravital and *ex vivo* imaging studies have shown that liver merozoite release, rather than occurring through simple rupture of the host hepatocyte, takes place through the release of membrane-bound ‘bags’ of merozoites (called extrusomes or merosomes). These are extruded into adjacent liver sinusoidal blood vessels to be swept into the bloodstream where they disintegrate beyond the liver to release their cargo (Tarun et al., 2006; Sturm et al., 2006; Baer et al., 2007). Merosome formation and liver stage merozoite egress, but not intracellular liver stage development, is prevented by genetic disruption or pharmacological inhibition of the parasite cGMP-dependent protein kinase PKG (see Box 2) (Falae et al., 2010; Govindasamy et al., 2016; Vanaerschot et al., 2020).

The first morphological indicator of liver merozoite egress is parasitophorous vacuole membrane (PVM) rupture, which may occur after a period of increasing permeability, although not all researchers observe this (Baer et al., 2007; Sturm et al., 2009). Ablation of the liver stage protein LISP1 had no effect on merozoite development but prevents PVM rupture (Ishino et al., 2009); however how LISP1 mediates its function is unknown. PVM rupture is followed by destabilisation of the host hepatocyte cell membrane and separation from its underlying cytoskeleton (Burda et al., 2017a), likely enabling merosome formation (see poster). In accordance with this model, analysis of merosome structure indicates that the merosome membrane is derived from the host cell plasma membrane (Graewe et al., 2011). Both PVM rupture and host membrane cytoskeletal changes that lead to merosome formation are inhibited by E64 (Burda et al., 2017a), a peptidyl epoxide selective for cysteine proteases. Importantly, very recent reverse genetic work has implicated the cysteine protease-like PbSERA4 (‘group II’ SERA; see Box 2) in liver stage egress (Putrianti et al., 2020). Expression and proteolytic processing of the ‘group III SERA’ (see Box 2) PbSERA3 has also been demonstrated in liver stage parasites (Schmidt-Christensen et al., 2008), although in this case its importance in egress was not examined. If SERA proteins are targets of E64 in exoerythrocytic parasites, this implicates these putative proteases in PVM rupture and possibly in downstream events. In further support of a role for proteolysis in liver stage egress, two separate studies have demonstrated that egress requires the serine protease SUB1 (see Box 2) (Tawk et al., 2013; Suarez et al., 2013), first implicated in asexual blood stage egress (see below). It is conceivable that, as in asexual blood stages, SUB1 activates the function of the SERA proteins in the liver egress pathway (see poster and below).

Unlike the host cell plasma membrane, the PVM has no underlying cytoskeleton, so whether or how it could be directly disrupted by protease activity is a mystery. Involvement of a third enzyme class in liver stage egress was convincingly demonstrated by disruption of the gene encoding the putative phospholipase PbPL, which localises to the PVM. This produced a defect in PVM rupture and delayed egress (Burda et al., 2015). As mentioned above, PbPL is also implicated in sporozoite egress from oocysts, but its substrate specificity and targeted structure(s) remain unknown (see poster).

## Egress of asexual blood stage merozoites

Despite some uncertainty in earlier work, it is now widely accepted that – as in the liver stage – blood stage merozoite egress comprises PVM rupture rapidly followed by rupture and vesiculation of the RBC membrane (RBCM) (the so-called ‘inside-out’ model). Several observations have added granularity to this picture, indicating that rupture of each membrane is preceded by a transient permeabilisation or ‘poration’ step.

As in the liver stage, PKG plays a key early role in asexual blood stage merozoite egress, as demonstrated with selective pharmacological inhibitors combined with gatekeeper mutagenesis, and more recently by conditional gene disruption (Taylor et al., 2010; Collins et al., 2013; Baker et al., 2017; Ressurreição et al., 2020; Koussis et al., 2020). Consistent with this, blockade of cGMP synthesis by conditional knockout of the guanylyl cyclase GCα blocks egress (Nofal et al., 2021), while pharmacological inhibition of phosphodiesterases (which degrade cGMP) conversely leads to premature egress (Collins et al., 2013), likely through dysregulated over-accumulation of cGMP. The parasite protein phosphatase 1 (PfPP1) plays an essential role in stimulating GCα activity, perhaps by regulating its capacity to respond to phospholipid-based signals (Paul et al., 2020). PKG activation results in rapid mobilisation of cytosolic Ca^2+^ levels that in turn activate a set of plant-like Ca^2+^-dependent protein kinases (see Box 2 and poster). Of these, CDPK5 is required for efficient egress, cooperating with PKG in a poorly understood manner to control the process (Dvorin et al., 2010; Absalon et al., 2018). Unlike gametocyte activation (see Box 1), blood stage merozoite egress is not obviously controlled by changing exogenous environmental signals, and the endogenous ‘trigger’ that initiates PKG activation in the mature schizont remains elusive. Recent intriguing data have implicated exogenous phosphatidylcholine as a putative egress stimulatory factor (Paul et al., 2020), but more research is needed to understand how access of this serum factor to the intracellular parasite might be temporally regulated.

Building on early descriptive work in avian and simian *Plasmodium* species (Trager and McGhee, 1956; Dvorak et al., 1975), the morphological transitions that lead up to merozoite egress have been more recently described in a series of EM and light microscopy studies, combined with the use of compartment- or membrane-specific fluorescent markers and pharmacological inhibitors to ‘trap’ otherwise transient steps in the pathway. Minutes before egress, the PVM alters in shape, rounding up without apparently swelling. As in the liver stage, this can be accompanied by increased PVM permeability (Hale et al., 2017), but this is not always observed (Glushakova et al., 2018). The segmented merozoites rearrange into a symmetrical ‘flower-like’ structure, before sudden rupture and vesiculation of the PVM, fragments of which form multilamellar vesicles within the enclosing RBCM (see poster). PVM rupture allows increased movement of the merozoites, as well as poration and collapse of the RBCM, rapidly followed by final RBCM rupture (Glushakova et al., 2005, 2009; Gilson and Crabb, 2009; Glushakova et al., 2010; Yahata et al., 2012; Collins et al., 2017; Thomas et al., 2018). Highspeed video microscopy has shown that RBCM rupture initiates at a single point, allowing the membrane to rip open while curling and everting, ejecting the merozoites (Abkarian et al., 2011) (see poster).

The use of pharmacological agents, fluorogenic probes and conditional mutagenesis have provided further insights into the regulation of individual steps in the pathway. A mechanistic role in egress has been proposed for a large parasite protein termed schizont egress antigen-1 (SEA1) (Raj et al., 2014), but very recent work has confirmed a previously suspected role in DNA replication instead (Perrin et al., 2021). PVM rounding and likely all downstream steps depend upon mobilisation of intracellular Ca^2+^ (Glushakova et al., 2013; Brochet et al., 2014), itself regulated by PKG activation. PKG activity and Ca^2+^ are also required for discharge and activity of SUB1 from merozoite secretory organelles, called exonemes, into the PV lumen (Yeoh et al., 2007; Agarwal et al., 2013; Withers-Martinez et al., 2014). There, SUB1 cleaves several soluble PV and merozoite surface proteins, including MSP1 (Yeoh et al., 2007; Koussis et al., 2009). Conditional knockout of *SUB1* produced an egress block indistinguishable from inhibition or knockout of PKG (Thomas et al., 2018), indicating a key role for SUB1 early in the pathway. SUB1-mediated cleavage of MSP1 appears to be important for efficient egress, perhaps due to interactions between MSP1 and the host RBC cytoskeleton (Das et al., 2015). RBCM rupture, but not RBCM poration or PVM rupture, is prevented by E64 and its more membrane-permeable analogue E64d, indicating a role for cysteine protease(s) in the final step of egress (Wickham et al., 2003; Glushakova et al., 2010; Chandramohanadas et al., 2011; Dans et al., 2020). A suggested role for the cysteine protease dipeptidyl peptidase DPAP3 was ruled out by knockout studies (Ghosh et al., 2018; Lehmann et al., 2018). Host RBC calpain-I (a Ca^2+^-dependent cysteine protease) was implicated in egress (Chandramohanadas et al., 2009; Millholland et al., 2013), but the essentiality of this was put in doubt by the observation that calpain-I-null mice support normal replication of *P. berghei* (Hanspal et al., 2002). Further light on likely targets of E64 and E64d was shed by the demonstration that SUB1 activates members of the SERA family (previously implicated in sporozoite egress from oocysts). In the case of *P. falciparum,* these include the ‘group IV’ SERA, PfSERA5, which plays a non-enzymatic role in regulating the kinetics and efficiency of egress (Stallmach et al., 2015; Collins et al., 2017), and the ‘group III’ SERA, PfSERA6 (the *P. falciparum* orthologue of PbSERA3), which has a distinct, essential role in RBCM rupture, probably by mediating proteolytic degradation of the RBC cytoskeletal protein β-spectrin (Ruecker et al., 2012; Thomas et al., 2018).

Based on work in the related parasite *Toxoplasma* (Kafsack et al., 2009), interest has focused on the potential role of pore-forming proteins in blood stage merozoite egress. The *Plasmodium* genome encodes five proteins with membrane attack complex/perforin (MACPF) domains, and PPLP1 and PPLP2 have been proposed to play roles in egress based on expression, localisation and inhibitor studies, as well as the demonstration that their recombinant forms possess RBC lytic activity (Garg et al., 2013; Wirth et al., 2014; Garg et al., 2020). However, this notion remains contentious, as genetic ablation of either PPLP1 or PPLP2 has no effect on asexual replication rates, or RBCM poration at egress (Deligianni et al., 2013; Wirth et al., 2014; Yang et al., 2017). The proteins may perform redundant and complementary functions, and further work is needed to clarify their essentiality in egress.

Collectively, the current data point to a blood stage merozoite egress model in which PKG together with CDPK5 triggers the intracellular release and activation of a number of effector molecules that disrupt the bounding membranes within minutes (see poster). This is in contrast to earlier suggestions that breakdown of the host cell cytoskeleton takes place slowly over many hours (Millholland et al., 2011). Perhaps in contrast to sporozoite egress, merozoite egress is a ‘passive’ process in that the merozoite actinomyosin contractile system, which drives subsequent invasion of a fresh RBC, is not required for egress (Das et al., 2017; Perrin et al., 2018).

## Egress of gametes

Gamete egress has been most closely examined in the rodent species *P. berghei.* Both male and female *P. berghei* gametocytes possess unique electron-dense cytoplasmic vesicles termed osmiophilic bodies (OBs); these are larger and more abundant in female *P. berghei* gametocytes, and completely absent from male *P. falciparum* gametocytes. In a process reminiscent of exonemes in blood stage merozoite egress, discharge of OBs into the PV occurs concomitant with egress (Kuehn and Pradel, 2010; Ishino et al., 2020), leading to the notion that OBs contain important molecules with possibly sex-specific role(s). However, gene disruption analysis provides conflicting data on this. Knockout of the gene encoding the first identified protein marker for OBs, G377, resulted in loss of OBs from female *P. falciparum* gametocytes and failure to infect mosquitoes (Suárez-Cortés et al., 2016). However, in contrast with earlier studies, this was not due to an egress defect, suggesting that OBs function post egress. Proteomic analysis of the OB-free mutants allowed the identification of several other OB proteins, including GEST, the subtilisin-like protease SUB2 and the dipeptidyl peptidase DPAP2 (Suárez-Cortés et al., 2016). Disruption of *DPAP2* partially reduced egress (Suárez-Cortés et al., 2016). Other reverse genetic studies revealed that several OB-located proteins, MDV1 (also known as PEG3) (Ponzi et al., 2009), GEST (Talman et al., 2011) and GEP (Andreadaki et al., 2020), are required for efficient egress. Disruption of the putative pantothenate transporter PAT, which localises to membranes of OBs and other parasite secretory organelles, also reduces egress (Kehrer et al., 2016). A further, non OB-located gametocyte ferlin-like protein called FLP is also required for egress (Obrova et al., 2019); like PAT (Kehrer et al., 2016), this protein likely has an indirect role, probably being required for the Ca^2+^-dependent exocytosis involved in discharge of OBs and other important secretory organelles (Coleman et al., 2018). The gametocyte TRAP family protein MTRAP, originally identified as a putative merozoite invasion ligand, has been implicated in gametocyte egress (Bargieri et al., 2016), while PMX is also required (Pino et al., 2017), perhaps due to its role in maturation of SUB1, which is implicated in male gamete egress (Pace et al., 2019). Further support for a role for protease activity in egress was provided by evidence that broad-spectrum small-molecule protease inhibitors prevent egress (Sologub et al., 2011); these include E64d, implicating at least one cysteine protease in egress. Although none have yet been definitely identified to be essential for gamete egress, by analogy with sporozoite and asexual parasite stages, it seems likely that a SERA family member is involved (Pace et al., 2019).

Similar to the other parasite developmental stages, time-lapse video light microscopy (sometimes combined with fluorescent reporter-expressing parasites) and EM have revealed the morphological transitions involved in gamete egress. In *P. falciparum,* the process initiates by ‘rounding up’ of the crescent-shaped cells, a process that is PKG dependent (McRobert et al., 2008) (see poster). *P. berghei* gametocytes undergo swelling (Andreadaki et al., 2018). Production of cGMP required for gametocyte activation and egress is mediated by the guanylyl cyclase GCα, but also depends on the associated polytopic membrane protein GEP1 (distinct from GEP discussed above), which is thought to bind more strongly to GCα in the presence of xanthurenic acid (XA) and thus enhance GCα-mediated cGMP synthesis (Jiang et al., 2020). Subsequent events, including activation of the Ca^2+^-dependent protein kinase CDPK4 (Billker et al., 2004), depend on Ca^2+^ mobilisation that is induced in a PKG- and phosphoinositide-specific phospholipase C (PI-PLC)-dependent manner (Martin et al., 1994; Brochet et al., 2014). Within minutes of activation, the OBs migrate to and fuse with the parasite plasma membrane (Kuehn and Pradel, 2010; Ishino et al., 2020), before PVM rupture and vesiculation take place, which appears to occur at multiple points (Sologub et al., 2011; Wirth et al., 2014; Andreadaki et al., 2018). In contrast, subsequent rupture of the RBCM initially occurs at a single site (Sologub et al., 2011; Andreadaki et al., 2018). Thus, as in the liver and asexual blood stages, gamete egress appears to follow the ‘inside-out’ model (see poster).

The precise roles in these membrane rupture events of most of the proteins mentioned above is unknown (see poster). Loss of GEP prevents axoneme motility in microgametes, but this is not its only role as macrogamete egress was also blocked in GEP-null mutants, perhaps because OB discharge was retarded or reduced (Andreadaki et al., 2020). As is the case for many malarial proteins, GEP has no recognisable protein domains and no orthologues outside the Apicomplexa, making functional prediction difficult. A greater mechanistic understanding has been gleaned in the case of the putative pore-forming protein PPLP2, a member of the family of *Plasmodium* perforin-like proteins referred to above. Ablation of PPLP2 produced a dramatic egress phenotype (male-specific in *P. berghei*), in which PVM rupture and vesiculation occurred normally, but the RBCM remained intact and unpermeabilised (Deligianni et al., 2013; Wirth et al., 2014; Andreadaki et al., 2018). This indicates a specific role for PPLP2 in RBCM rupture, presumably through the formation of pores that weaken the membrane. In support of this, artificial selective permeabilisation of the RBCM with exogenously-applied equinatoxin II leads to the release of PPLP2-null mutant parasites (Deligianni et al., 2013; Wirth et al., 2014), and recombinant PPLP2 has been demonstrated to lyse RBCs (Wirth et al., 2014). Reminiscent of SUB1 regulation in asexual blood stage egress, PPLP2 is thought to be discharged just prior to egress from vesicular structures distinct from OBs (Wirth et al., 2014). This discharge is reduced in mutants lacking a patatin-like phospholipase, which also show an egress defect (Singh et al., 2019). Intriguingly, the protease inhibitor studies mentioned above showed that the chymotrypsin inhibitor TLCK prevents RBCM rupture without preventing its permeabilisation (Sologub et al., 2011), indicating a requirement for a protease-mediated step independent of PPLP2 activity. Although many questions remain unanswered regarding gamete egress, these fascinating insights imply that – just as with merozoite egress – distinct mechanisms govern PVM rupture, RBCM poration and RBCM rupture.

## Conclusions and future perspectives

We hope that it is evident from this brief summary that there are clear distinctions between the biological features and molecular pathways operating to mediate egress at the different developmental phases of the *Plasmodium* life cycle. At the same time, there is strong evidence for a central essential role for PKG in liver stage, asexual blood stage and gamete egress, and for SERA family members in all egress stages. This suggests underlying commonalities that indicate evolutionarily convergent strategies. Despite recent insights gained largely from improvements in conditional genetics in *Plasmodium*, key questions remain regarding the regulation of and effector molecules involved in egress, and whether egress can be a target of new types of chemotherapeutics (see poster and ‘Key questions‘). Current evidence suggests that at least PKG could represent an exciting drug target (Baker et al., 2020). Given the continued interest in all these crucial phases of the *Plasmodium* life cycle, we are confident that the answers to many of these questions will soon become clear.

## Box 1 The malaria parasite life cycle

Male and female gametocytes in a blood meal are rapidly activated in the mosquito midgut through a combination of lowered temperature relative to the vertebrate host and the presence in the mosquito midgut of a vector-derived soluble factor called xanthurenic acid (XA). The males undergo three rounds of mitosis over the course of ~10 min to form eight flagellated microgametes, whereas the females form a single macrogamete. Gamete egress and fertilisation generates zygotes which transform into motile ookinetes. These migrate through the midgut epithelium, arresting beneath the basal lamina where they round up to form an oocyst. Over the next 10–15 days, hundreds to thousands of sporozoites develop within each oocyst. The oocyst then ruptures, releasing the sporozoites into the insect main body cavity (haemocoel) from where they are carried by the circulating haemolymph to the salivary glands.

Infection of the vertebrate host begins with inoculation of salivary gland sporozoites into the dermis during the bite of an infected mosquito. The sporozoites migrate via the bloodstream to the liver where they invade hepatocytes and develop over the course of 2–10 days (depending on the *Plasmodium* species) to form large, multinucleated liver stage schizonts, which undergo segmentation to form merozoites. This is a huge amplification step as each viable sporozoite can generate up to ~30,000 liver stage merozoites (Baer et al., 2007; Sturm et al., 2009; Burda et al., 2017b). These are released into the bloodstream, rapidly invading RBCs to initiate the asexual erythrocytic cycle. The intracellular parasite develops and replicates, digesting the host cell haemoglobin (over ~48 h in the case of *P. falciparum*) and eventually forms a multinucleated schizont that produces 16 or more daughter merozoites. These burst out of the host RBC and rapidly infect new RBCs to repeat the cycle of invasion, replication and egress, resulting in exponential expansion of the parasite population. Whereas the liver stages of malarial infection are clinically silent, the asexual blood stage cycle is responsible for all the manifestations of disease.

During each asexual growth cycle, a subset of parasites take an alternative developmental route and commit to sexual development to produce gametocytes, the forms required for transmission to the mosquito vector. In all three intracellular stages in the vertebrate host, parasite development occurs within a membrane-bound parasitophorous vacuole (PV).

## Box 2 The PKG–SUB1–SERA pathway – a universal route to malarial egress?

The parasite protein kinase PKG plays a key signalling role in several important transitions throughout the life cycle. PKG is activated by the cyclic nucleotide cGMP, so regulation of levels of this intracellular second messenger is central to control of egress; indeed, the timing of egress may be governed simply by cGMP accumulating in the parasite until it reaches a critical intracellular threshold concentration required to fully activate PKG. This is likely achieved by a balance between cGMP synthesis by guanylyl cyclases (GCs) and cGMP degradation by phosphodiesterases (PDEs). *Plasmodium* PKG has three cGMP-binding sites arranged surrounding the central kinase domain in an architecture that may allow the transfer of conformational rearrangements as the sites become occupied (Deng et al., 2003; Kim et al., 2015; Franz et al., 2018; El Bakkouri et al., 2019). PKG activation results in production of another second messenger inositol (1,4,5)-trisphosphate (IP3) and mobilisation of intracellular Ca^2+^ (Brochet and Billker, 2016). This activates plant-like Ca^2+^-dependent protein kinases (CDPKs) and triggers discharge of specialised secretory organelles (see, for example, Collins et al., 2013; Absalon et al., 2018).

One secretory organelle resident common to several parasite life cycle stages is the subtilisin-like parasite serine protease SUB1, which is critical for merozoite (both liver and blood stage) and gamete egress (see main text). Maturation of SUB1 is mediated by the aspartic protease plasmepsin X (PMX) (Pino et al., 2017; Nasamu et al., 2017). Maturation of PMX is in turn dependent upon an endoplasmic reticulum-resident CREC family Ca^2+^-binding protein (PfERC), (Fierro et al., 2020).

SUB1 activates the function of a set of proteins of the serine repeat antigen (SERA) family. All SERAs possess a central domain homologous to papain-like proteases (clan CA, family C1; https://www.ebi.ac.uk/merops/) and are divided into four subfamilies (‘groups I—IV’) (Arisue et al., 2011; Arisue et al., 2020). The SERAs may mediate egress throughout the parasite life cycle, albeit via different mechanisms (see main text). Of particular interest is PfSERA6 (the ‘group III’ SERA; the *P. berghei* orthologue is PbSERA3), which is implicated in both liver and blood stage egress. The ‘group I’ SERA PbSERA5 (egress cysteine protease 1 or ECP1) is required for sporozoite egress but appears to lack SUB1 cleavage sites, suggesting a SUB1-independent activation mechanism.

## Key conceptual questions regarding malaria parasite egress

- What is the molecular makeup of the PVM and oocyst wall and how are they degraded?
- What are the egress triggers in the different life cycle stages and how are these signals sensed and transduced?
- What additional egress effectors have yet to be identified?
- Can signalling molecules and effector molecules involved in egress be targets of clinically efficacious therapeutic or prophylactic antimalarial drugs?
